# Direct growth inhibition assay of total airborne fungi with application of biocide-treated malt extract agar

**DOI:** 10.1016/j.mex.2015.07.002

**Published:** 2015-07-20

**Authors:** Chin Ming Er, N.M. Sunar, A.M. Leman, N. Othman

**Affiliations:** a Department of Water and Environmental Engineering (DWEE), Faculty of Civil and Environmental Engineering (FKAAS), Universiti Tun Hussein Onn Malaysia, 86400 Parit Raja, Batu Pahat, Johor, Malaysia; b Department of Chemical Engineering Technology, Faculty of Engineering Technology (FTK), Universiti Tun Hussein Onn Malaysia, 86400 Parit Raja, Batu Pahat, Johor, Malaysia

**Keywords:** Direct growth inhibition assay of airborne fungi, Indoor air quality, Airborne fungi contamination, Biocide, Fungal growth inhibition, Potassium sorbate, NMAM 0800

## Abstract

Indoor air pollution by airborne fungi has risen to become a common issue all over the world and it is hazardous to indoor occupants’ health as it is associated with a series of respiratory-related and skin-related diseases. Selected bioactive compounds from the food industry have been suggested to be effective against individual fungus isolated from indoor environment. However, the techniques used to evaluate these compounds were lengthy and unsuitable against total airborne fungi. Therefore, this paper describes an assay to assess the effectiveness of a bioactive compound to inhibit growth of total airborne fungi.

• A combination and modification of previous methods and the NIOSH Manual Analytical Standard Method (NMAM 0800) is proposed.
• This method concurrently samples the total airborne fungi and evaluates the ability of bioactive compounds (potassium sorbate in this paper), as a biocide, to treat these indoor airborne fungi.
• The current method shortens the time of evaluation from 30 days to only 5 days and employs the counting of colony forming units (CFUs) to ease the measurement of the growth of fungi.

## Graphical abstract

**Figure:**
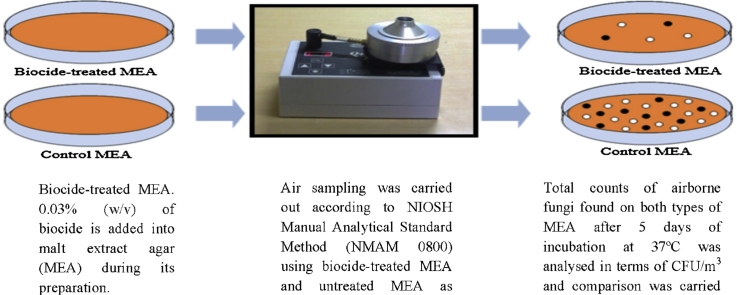

## Method details

### Study background

Indoor airborne fungi contamination has became a serious issue in indoor air quality (IAQ) management as it is correlated with various diseases such as damage of the respiratory tract that involves nose and lung, skin infection, mucous membrane irritation and a series of symptoms classified under the sick building syndrome [1]. The effect of conventional fungicides used in disinfecting the environment is not long-lasting [2] and might be toxic to humans. Besides, microorganisms have developed resistance against existing fungicides [3]. As the conventional fungicides are not suitable for indoor usage, more environmental friendly compounds that are non-toxigenic to humans are required [4]. A few biocides used in the food industry have been evaluated and shown to be effective against isolated indoor waterborne fungi [4], [5], [6], [7], [8], [9] and single isolated indoor airborne fungus [9]. Nevertheless, the techniques used in the previous method focused on evaluation of bioactive compounds against single isolated fungus by taking into consideration that the fungus growth is measured by diameter of fungus colony, which is very hard to measure, and required 30 days to accomplish [4]. While, the previous NMAM 0800 method is meant for bioaerosol sampling only [10]. Thus, these previous methods were not suitable for evaluation of growth inhibition of total airborne fungi. Hence, a method to evaluate the performance of bioactive compounds (potassium sorbate in this study), as a biocide [11], [12] in growth inhibition of total airborne fungi was reported here.

The biocide's antifungal activity was assessed by a direct growth inhibition assay of total airborne fungi that comprises the air samplings of total airborne fungi, incubation and enumeration of fungal colonies formed. Biocide-incorporated and untreated control MEA were used in these procedures. The assay takes into account that the total number of the viable fungi can be indicated by colony forming unit (CFU) analysis [13].

### Preparation of potassium sorbate-incorporated malt extract agar

Firstly, 0.03% (w/v) of potassium sorbate was incorporated into malt extract agar (MEA). The mixtures were sterilized in an autoclave at 121 °C for 15 min. Pre-sterilized Petri dishes measuring 90 mm × 15 mm were filled with 20 mL of the biocide-treated MEA under aseptic conditions. The solidified biocide-treated MEA plates were sealed with Parafilm. The control MEA plates without potassium sorbate were prepared under the same conditions. The whole process was carried out in a laminar flow hood.

### Air sampling of total airborne fungi

A single-stage viable cascade air sampler (SKC, USA) was fixed on a sample pump (SKC, USA). The attachment was calibrated before each usage using a rotameter. It was operated at a flow rate of 28.3 L/min as per requirement of Malaysia National Institute of Occupational Safety and Health (NIOSH) specified in a standard method, NMAM 0800 [10]. The air sampler was positioned at a height of 1.0–1.5 m from the floor at the midpoint of each testing area. The air sampling of the total airborne fungi onto the MEA plates was carried out for 5 min for each sample. Before the first air sampling and between every two consecutive measurements, the air sampler was cleaned with 70% ethanol to avoid contamination. Air sampling was carried out in triplicate onto the biocide-incorporated MEA and untreated control MEA, respectively. After the field sampling for 5 min, the plates were removed from the sampler, sealed with Parafilm and immediately placed in a cooler box with an ice pack at 4 °C to inhibit microbial growth. The air samples were then transferred to laboratory aseptic conditions within 2 h. All air samplings at a particular testing area were performed on the same day. The airborne fungi samples were cultured at 37 °C for 5 days.

### Viable counts of total airborne fungi

**Fig. 1:**
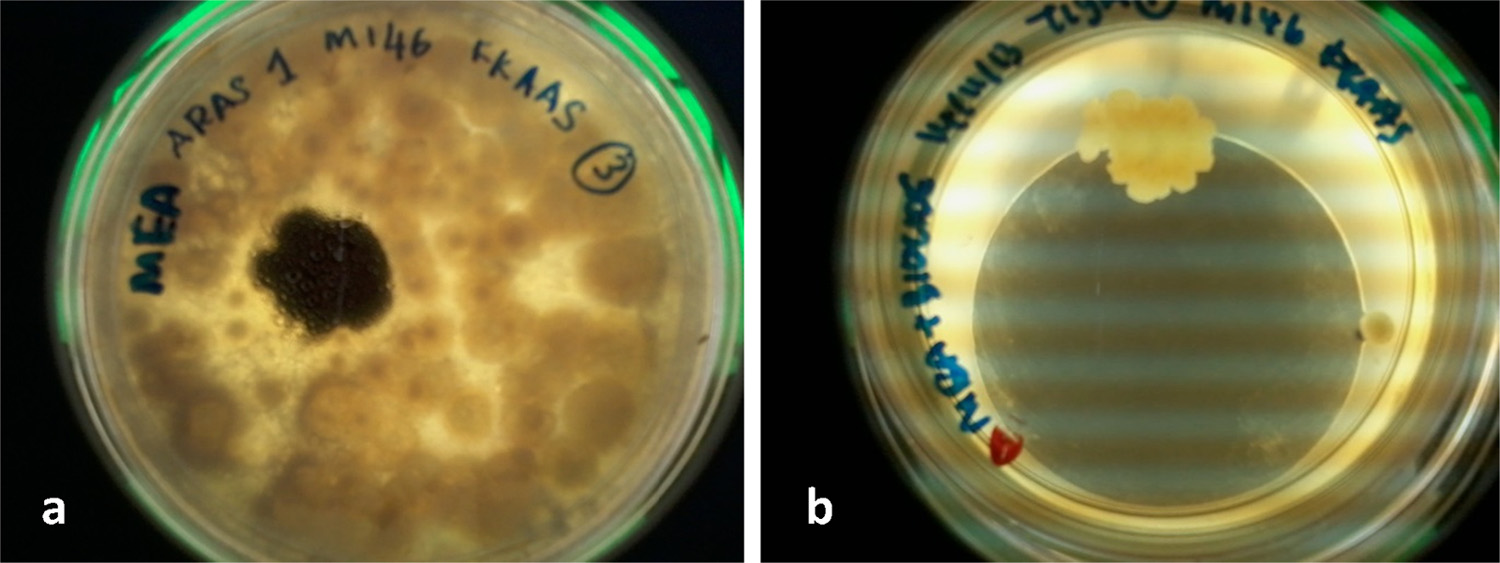
The comparison of the total airborne fungi found on (a) untreated MEA (control) and (b) biocide-treated MEA at a testing site in a building after 5 days of incubation at 37 °C.

The enumeration of the samples is indicated by colony forming unit (CFU) analysis [13]. The counting process was done by mounting the agar plate on digital colony counter and the colonies were counted manually (Fig. 1). The total number of the fungi colonies formed on the agar plate was then divided with the total volume of air drew by the sampler. The calculation is as follows [14]:

(1) $${\text{CFU/m}}^{3}=\frac{1000\;(\text{Number of colony})}{\text{Sampling time}\;(\min)}\times \text{Flow rate}\;(\text{L}/\min)$$

The fungal colonies formed were observed and counted daily until the fifth day to identify the colonies, to ensure no growth of bacterial colonies and to solve the difficulties in recognizing and counting the colonies. Since replicate samples were collected, the data was averaged.

### Biocide's antifungal activity toward total airborne fungi

The biocide inhibitive performance was determined by calculating the percentage of the reduction of the average total counts of the viable airborne fungi found on both types of agar plate, as shown in the equation below:

(2) $$\text{Biocide inhibitive performance}=\left[\frac{X-Y}{X}\right]\times 100\%$$

where, *X* is the average total counts of airborne fungi found on the control MEA, and *Y* is the average total counts of airborne fungi found on the biocide-treated MEA.

It was shown that, with this method, the total airborne fungi can grow on both types of MEA but with different total fungi counts. Moreover, consistent total counts of fungi can be found on the triplicates of sampling using the same type of MEA. Therefore, with this tabulation, the ability or effectiveness of a biocide (potassium sorbate in this study) against general microenvironment of the total airborne fungi at the testing site can be determined. This determination is important as it provides a new eco-friendly alternative to circumvent indoor air pollution by airborne fungi and therefore to provide a safe and comfortable indoor environment with good indoor air quality. In this study, potassium sorbate was shown to effectively reduce the total counts of airborne fungi with around 84% of biocide inhibitive performance (Table 1).

## Additional information and recommendations

The incubation temperature of 37 °C was used in this study to selectively sample fungi that are pathogenic to humans [15]. However, a more common and lower incubation temperature, such as 25 °C could be used for general purposes. A 5 days incubation period was used according to the standard method, NMAM 0800 [10] and previous indoor airborne fungal sampling studies [16], [17], [18]. It is a standard incubation period for fungi samples in indoor air quality studies.

Potassium sorbate was used as an example of the subject of the assay in this study because of its previous performance in controlling the growth of individual indoor fungus [4], [5], [6], [7], [8], [9]. However, this method could be used to assess the biocide inhibitive performance of other new alternatives/bioactive compounds against the total indoor airborne fungi.

## Figures and Tables

**Table 1:** The biocide inhibitive performance of the biocide, potassium sorbate against total indoor airborne fungi.

Averaged total counts of airborne fungi on control MEA, CFU/m^3^ (*n* = 3)	Averaged total counts of airborne fungi on biocide-treated MEA, CFU/m^3^ (*n* = 3)	Biocide inhibitive performance (%)
269	42	84.4
